# Suberin. A component of citrus peel extracts

**DOI:** 10.1002/jsfa.70075

**Published:** 2025-08-08

**Authors:** John A. Manthey, Kristen A. Jeffries

**Affiliations:** ^1^ US Horticultural Research Laboratory Agricultural Research Service Fort Pierce FL USA

**Keywords:** citrus peel, ultrafiltration, HPLC baseline, LH20 chromatography, suberin

## Abstract

**BACKGROUND:**

Flavanone glycosides have been traditionally recovered from citrus peels and commercialized as herbal supplements. Commercial recoveries of these citrus peel flavonoids typically involve initial water extractions, and in certain cases the removal of pectin by ion‐exchange resins. A consistent feature of high‐performance liquid chromatography (HPLC) chromatograms of such aqueous peel extracts is significantly elevated broad baselines. These baselines suggest the occurrence of wide populations of possibly similar chemical entities. In this study, isolations of these materials by either ultrafiltration or LH20 column chromatography led to the recoveries of fractions, termed ‘baseline fractions’ particularly enriched in the peel materials responsible for these elevated baselines. The goal of this study was to isolate the material responsible for these elevated baselines and to conduct an initial chemical characterization and possible identification of this portion of citrus peel water extracts.

**RESULTS:**

The water‐soluble component of these baseline fractions was determined to be protein as determined by its Fourier‐transform infrared (FTIR) spectroscopy. The remaining major components of these baseline fractions were freely soluble in methanol, and the FTIR and nuclear magnetic resonance (NMR) spectra of these components were indicative of the plant polymer, suberin. Size exclusion chromatography showed this material to largely occur between 10 and 60 kDa as would be expected for this plant polymer.

**CONCLUSION:**

It is proposed in this study that suberin is responsible for the broad elevated HPLC baselines of citrus extracts. Furthermore, this multifunctional chemical cell wall material may possibly contribute to the overall biological effects attributed to flavanone‐enriched citrus peel supplements, in which this material frequently occurs. Published 2025. This article is a U.S. Government work and is in the public domain in the USA. *Journal of the Science of Food and Agriculture* published by John Wiley & Sons Ltd on behalf of Society of Chemical Industry.

## INTRODUCTION

Citrus peels are rich in numerous potentially health‐benefiting compounds, including primarily flavanone glycosides, hydroxycinnamates, and polymethoxylated flavones.^1^, ^2^ Large scale recoveries of these compounds usually involve initial aqueous peel extractions (creating ‘peel juice’), a material similarly produced as a by‐product in juice production.^3^ Peel juice and molasses are composed of soluble sugars (60–70% of the dry weight), suspended tissue fragments, non‐dialyzable solids (proteins), organic acids, mineral ions, phenolic compounds, polyols, and other components such as limonoids.^4^ Peel juice may be subsequently passed through an anion‐exchange resin to recover and thus remove a significant amount of the pectin typically in these peel extracts. These pectin‐depleted solutions can then be passed through adsorbent resins to remove free sugars and other cell wall polysaccharides in the column washes and to ultimately recover the many different peel phenolics by ethanolic washes of the exposed resins.^4^, ^5^ At this stage the ethanolic washes are typically highly enriched in many of the different low molecular weight peel phenolics and in other more lipophilic compounds. This material also appears to contain substantial amounts of other less well studied peel components, as made evident by highly elevated baselines observed in the analytical high‐performance liquid chromatography‐ultraviolet (HPLC‐UV) chromatograms of these adsorbent resin‐recovered materials. Analytical HPLC chromatography is a tool to separate and identify the chemical components of a sample.

Initially, these elevated HPLC baselines were hypothesized to arise from the occurrence of many different phenolic conjugates created as a result of mixed peroxidase/oxidase/laccase free radical coupling.^6^, ^7^, ^8^ A more recent novel hypothesis is that this baseline material consists mainly of a hugely diverse population of suberin‐like compounds. Suberin is an important structural component of plant cell walls, which plays critical roles in plant growth and development.^9^ This biopolymer occurs as polyesters of diverse aliphatic long chain fatty acids and glycerol, with domains of hydroxycinnamic acids and derivatives thereof.^9^, ^10^, ^11^, ^12^, ^13^ Due to the high degree of diversity in the monomers, only a general description of the chemical structure can be described. Polyfunctional long‐chain fatty acids typically with midchain functional groups including α,ω‐bifunctional aliphatic C16 to C26 fatty acids and in certain cases similarly long‐chain monofunctional fatty acids (up to C30) occur covalently linked with glycerol and terminal and/or mid‐chain functional groups. Included in these functional groups are polyaromatics, mainly ferulic acid and to a lesser degree tyramine, a mid‐chain unsaturated methylene C=C, and partly hydrolyzed epoxides and hydroxyls at this latter location.^14^ Suberin from several different plant sources have been shown using gel permeation chromatography (GPC) to be distributed over a wide range of sizes.^15^, ^16^, ^17^, ^18^ Studies have shown that suberin is comprised of two main covalently linked domains, including a high molecular weight polyaliphatic component and a polyaromatic region.^16^, ^19^, ^20^ The average molecular weights determined from the corresponding chromatograms of native whole suberin and the chemically‐separated phenolic component were 44 and 27 kDa, respectively.^16^

Yet, regardless of its origin or composition, the presence of this material representing an undescribed peel component commonly occurring in citrus peel flavonoid supplements consistently lowered the percent composition of commercially recovered citrus peel flavonoids, particularly the flavanone glycosides hesperidin and narirutin in orange and mandarin peels, naringin in grapefruit peel, and eriocitrin in lemon and lime peels. There have been efforts towards valorization of citrus peel waste^21^, ^22^, ^23^, ^24^ and the healthful components of citrus have been studied for decades,^25^ but less is known about possible impacts of suberin on human health.^26^ Although speculative, it is feasible that suberin monomers could be bioactive in flavanone‐enriched citrus peel supplements. This study presents a preliminary characterization of the citrus peel materials responsible for elevated HPLC‐UV chromatogram baselines from citrus peel extracts using Fourier‐transform infrared (FTIR) and proton‐nuclear magnetic resonance (^1^H‐NMR) spectroscopy.

## MATERIALS AND METHODS

### Materials

Lemon and orange peel samples were commercially produced from clarified water extracts treated with anion exchange resins for the purpose of pectin recovery and subsequently with adsorbent resins for the recovery of the peel phenolic constituents – mainly the flavonoids. These compounds were recovered in 100% ethanol washes of the adsorbent resins and evaporated to dryness using a rotary evaporator. In this study, these samples are termed processed extracts of orange or lemon peels. In order to explore whether the citrus source affects the observed elevated HPLC baseline, a separate commercial sample of mid‐season orange peel molasses (54 °Brix concentrated aqueous peel extract) was included in this study. This molasses sample was diluted ten‐fold with water and the insoluble solids resulting from this water dilution were allowed to settle overnight. The upper portion of this partially clarified diluted sample was decanted and centrifuged at 10 000 × *g* for 30 min. This diluted molasses sample was further clarified by vacuum filtration through Whatman 1.5 μm micro fiberglass filters.

### Ultrafiltration

Ultrafiltration was performed using 3 kDa cut‐off Amicon ultra centrifugal filters obtained from EMD Millipore (P/N UFC5003). Small volumes of the earlier clarified water extracts (0.5 mL) were applied to the upper compartment of the centrifugal units, placed in a microfuge (Symphony 2417R; VWR International, Radnor, PA, USA) and spun at 14 000 × *g* for 20 min at room temperature.

### High‐performance liquid chromatography (HPLC)

Chromatographic analyses of samples were achieved by using a Waters 2695 HPLC coupled to a 996 photodiode array detector (Waters, Milford, MA, USA). HPLC separations with a 10 μL injection volume were achieved with a Waters XBridge C8 analytical column (4.6 mm × 150 mm) held at room temperature using a two solvent gradient composed initially of aqueous formic acid (0.5%)/acetonitrile (90/10 *v/v*) then increased in acetonitrile contents in linear gradients to 80:20 by 10 min, to 75:25 by 15 min, to 60:40 by 23 min, and 30:70 by 40 min, and retained at 30:70 to 53 min at a flow rate of 0.75 mL min^−1^. Eluted peaks were detected at 320 and 285 nm with UV spectra recorded between 240 and 400 nm. Software used to analyze data was Agilent ChemStation (version C.01.06).

Size exclusion chromatography (SEC) HPLC was performed using a TOSO TSKgel Alpha‐3000 column 7.8 mm × 30 cm, 7 um particle size. SEC HPLC was run at room temperature using a Varian ProStar 210 Solvent Delivery Module pump connected to a Varian ProStar 410 autosampler with a 10 μL injection volume. Elution conditions included the use of deionized water run at 1 mL min^−1^ for 30 min, followed by 20 min column washes with linear gradient of acetonitrile to 70%. Eluant was monitored at 200, 285, and 330 nm using a Jasco photodiode array (PDA) detector MD‐1515 (Jasco, Tokyo, Japan). The molecular weight calibration of the TSK gel column was performed with GPC/SEC calibration standards included in Agilent EasiVial PEG/PEO (P.N. 2080‐0201). Software used to analyze data was Jasco ChromNav (version 1.17.01).

### Preparative column chromatography

Column chromatography was run in a glass column (5 cm × 30 cm, Biotage, LLC, Charlotte, NC, USA) containing LH20 resin (Sigma‐Aldrich, St Louis, MO, USA) equilibrated in 100% methanol. Aqueous extracts were initially evaporated to dryness and subsequently dissolved in 100% methanol. The methanolic extracts were run on the LH20 column in methanol at 5 mL min^−1^ using a Biotage Horizon flash chromatography apparatus. Eluant was monitored at 285 and 320 nm in 20 mL fractions. Software integrated with the Biotage flash chromatography instrument was used for data analysis.

### Spectroscopy

FTIR spectra were recorded using a Perkin Elmer Spectrum One with integrated software. Samples were dried on International Crystals Laboratories (Garfield, NJ, USA) potassium bromide (KBr) cards for analysis. Infared spectra were recorded at room temperature with a scanning range of 4000–450 cm^−1^. The ^1^H‐NMR spectra were recorded of samples reconstituted in deuterated dimethyl sulfoxide (DMSO‐*d*_6_). The ^1^H‐NMR spectra were recorded with a Nanalysis 60 MHz spectrometer with a range of 10–0 ppm.

## RESULTS AND DISCUSSION

### High‐performance liquid chromatography (HPLC) baselines

**Figure 1:**
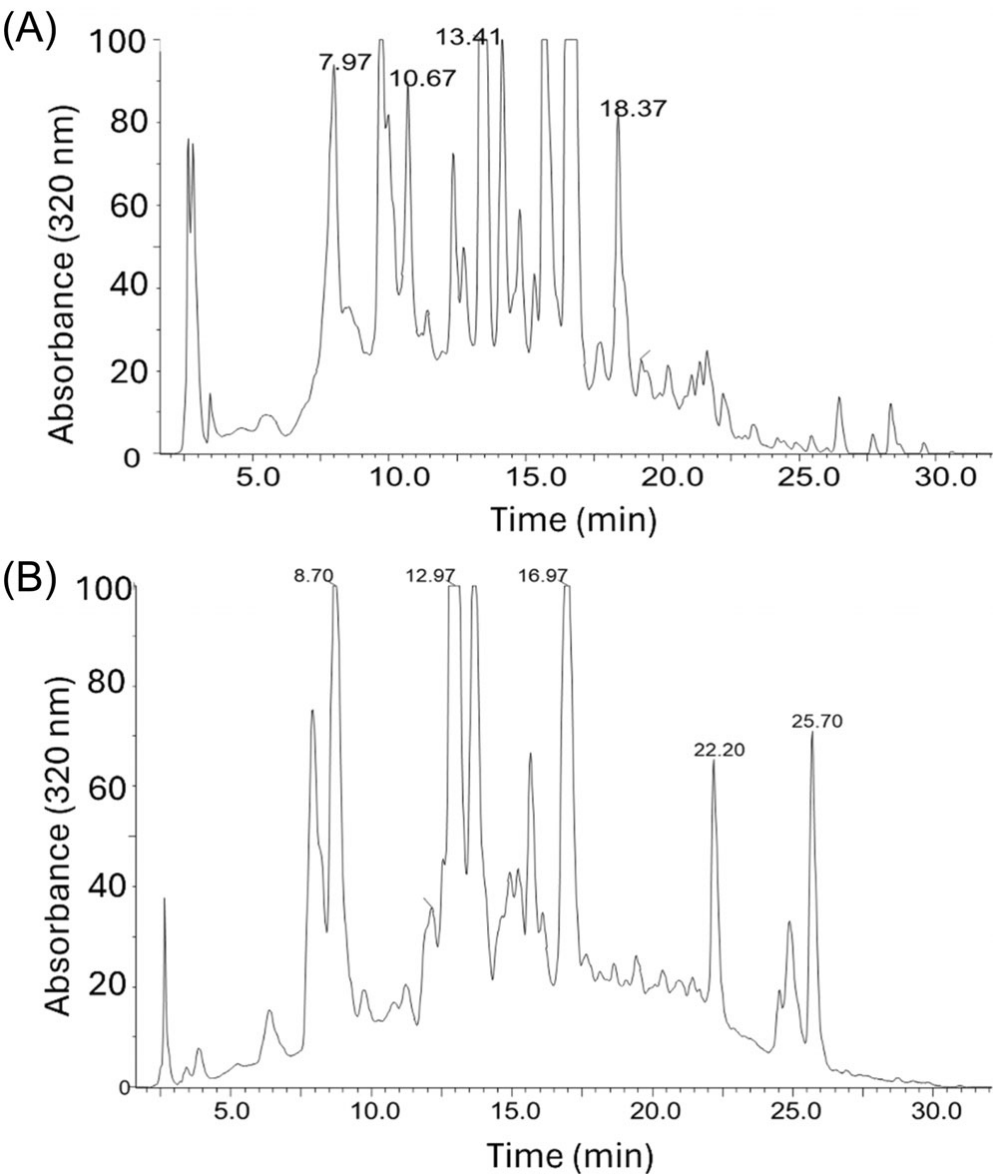
HPLC‐UV (320 nm) chromatogram of 10× water diluted clarified commercial orange peel molasses (A). HPLC‐UV (320 nm) chromatogram of the original processed dried lemon peel extract dissolved in water (B).

While the main chemical components of citrus peels, including cell wall polysaccharides, organic acids, sugars, terpenoids and many of the distinctive polyphenols, have been thoroughly characterized,^3^, ^4^, ^27^, ^28^, ^29^ other minor components remain less well studied. One interesting and unresolved feature of citrus peel extracts is their consistently observed broad elevated HPLC baselines, which are shown in Fig. 1(A),(B) for orange and lemon peels, respectively. This phenomenon is not only observed with C8 column chromatography as shown in Fig. 1, but also with other reversed phase chromatography, such as C16 and C18 columns (data not shown). Additionally, the citrus source did not impact the presence of the elevated baselines as they were observed in chromatograms of not only orange peel, lemon peel, and orange molasses, but also in those of other citrus varieties such as grapefruit, bergamot, sour orange, and tangerine (data not shown). The basis for such commonly observed elevated baselines has remained in question, but it is reasonable to suspect that the presence of large populations of related structures, such as those of the plant cell wall polymers lignin and suberin, might be responsible.^30^ It is also possible that considering the relatively large integrated areas under the HPLC baselines illustrated in Fig. 1, these peel components may represent appreciable amounts of the total solids in these citrus peel water extracts. In fact, as rough approximations, the sum totals of the individual peaks in Fig. 1 account for only 53% and 55% of the total 320 nm integrated areas (sum total of individual peaks plus additional areas under the baselines) for orange and lemon peels, respectively. Isolated baseline fractions (discussed later) were run on analytical HPLC, and when the ratios of the total peak areas of the baseline chromatograms were divided by the amount of material injected (in micrograms) onto the column and then compared to the same ratio from a hesperidin standard, the results indicated that the below‐the‐baseline material is the main component (approximately 90%) of the commercial processed aqueous extracts of citrus peels analyzed in this study.

### Molecular size

**Figure 2:**
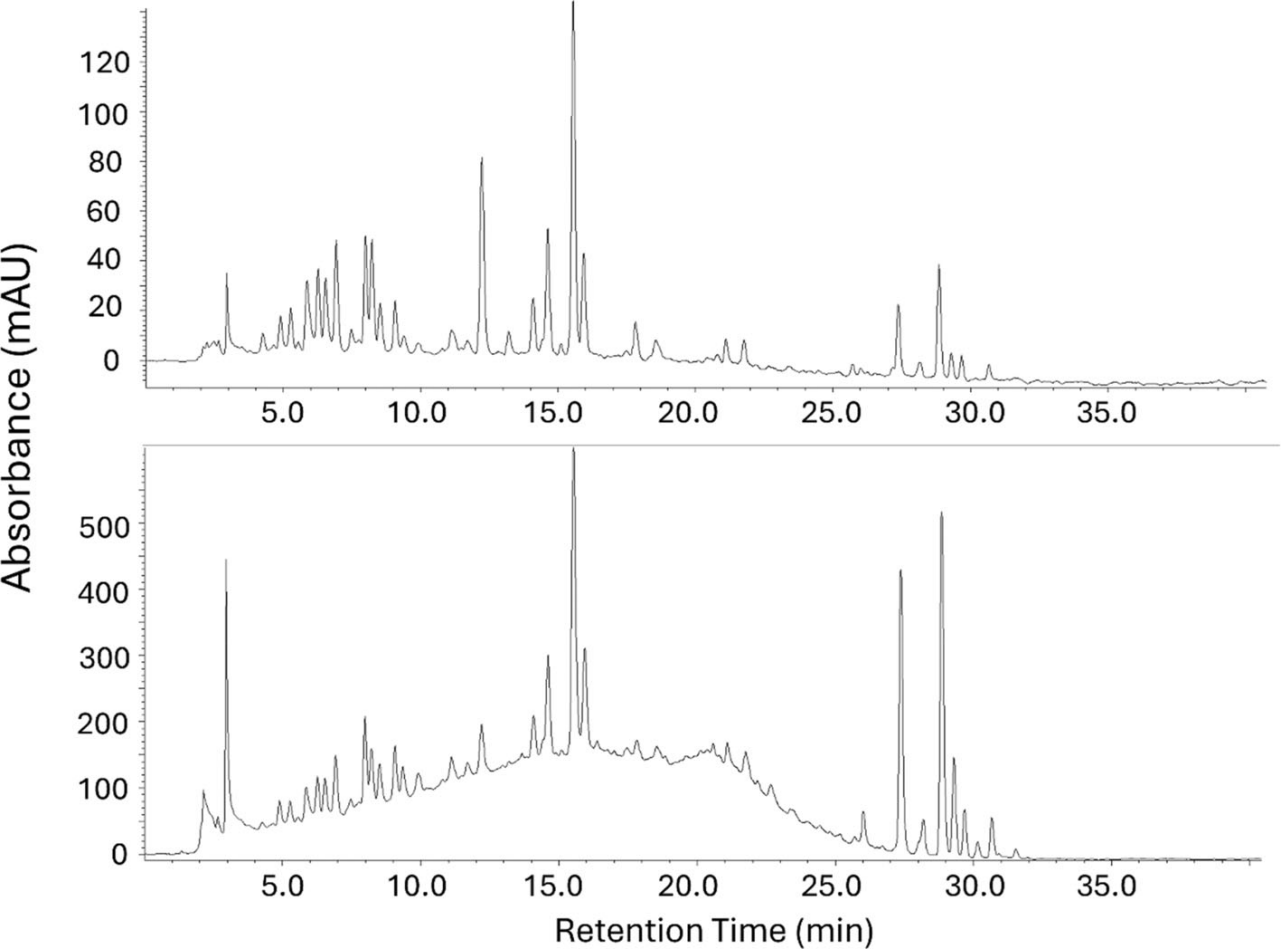
HPLC‐UV (320 nm) chromatogram of the clarified diluted orange peel molasses run through Amicon 3 kDa centrifugal filter. (Top) Permeate and (Bottom) retentate.

As an initial step in determining the chemical composition of the elevated baselines, components of different processed peel extracts were analyzed for their approximate molecular weight using several size exclusion and ultrafiltration techniques. As an initial technique, components of the processed citrus peel extracts were separated by ultrafiltration with an Amicon 3 kDa cut‐off ultracentrifugal filter designed to produce permeates enriched in compounds with molecular weights of less than 3 kDa, and retentates enriched with higher molecular weight components. Examples of the resulting HPLC baselines of the retentate and permeate obtained from using this technique with orange peel molasses are shown in Fig. 2, and from this example it is clear that the bulk of the high baseline material was present in the high molecular weight retentate. While individual lower molecular weight compounds occurred in the permeate, as expected, a large amount of low molecular weight compounds also occurred in the retentate. The reason for this is not clear, but it is possible that many citrus peel polyphenolics bind to components in the retentates. Many examples of binding of flavonoids to proteins have been reported,^31^, ^32^ and the binding of flavonoids to the materials used in ultrafiltration filters and membranes may also require investigation.

**Figure 3:**
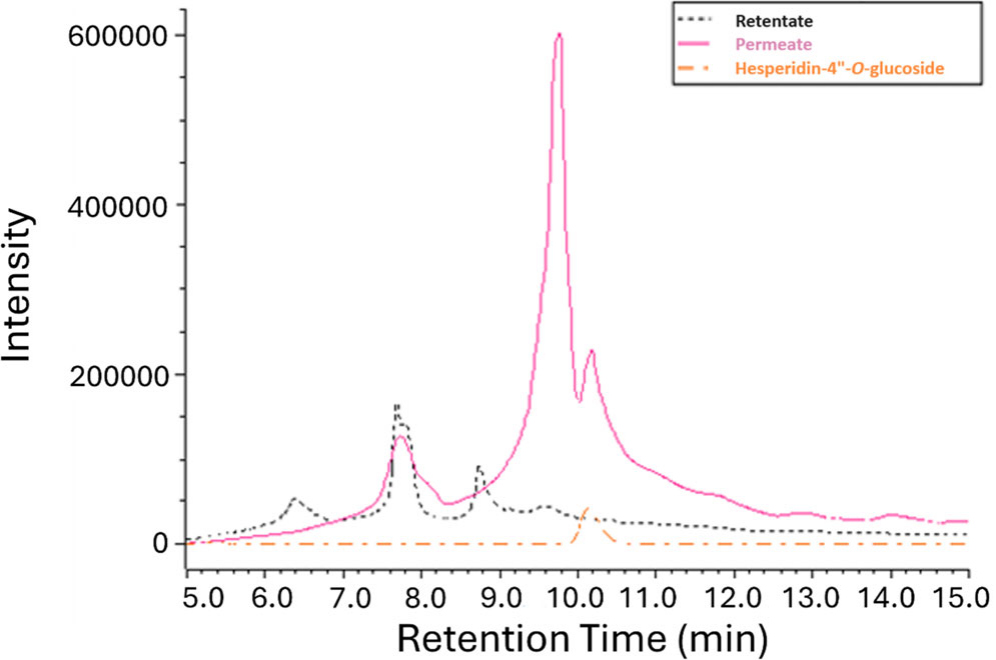
SEC chromatogram measured at 280 nm of diluted, clarified commercial orange peel molasses 3 kDa retentate and permeate, and the low‐molecular weight standard hesperidin‐4″‐*O*‐glucoside.

Differences in the relative molecular weights of the components in similarly obtained ultrafiltration permeates and retentates of the commercially supplied processed lemon peel extract were analyzed in greater detail by TSK Alpha‐3000 SEC. HPLC elution times of a series of polyethylene glycol molecular weight standards run on this column were measured and the data plotted as a smooth curve (Supporting Information Fig. S1). As shown in Fig. 3, components of an orange peel retentate eluted as three main peaks at 6.4 min (~40 kDa), 7.7 min (~5 kDa), and 8.8 min (~1.2 kDa), consistent with a previous report in which native whole suberin was 44 kDa.^16^ In contrast, the permeate contained much higher proportions of compounds at later elution times, with the last peak at 10.2 min, closely matching that of a molecular weight standard, hesperidin‐4″‐*O*‐glucoside (C_34_H_44_O_20_, 772 g mol^−1^). The polyethylene glycol molecular weight standards are likely to differ in the exact natures and degrees of interactions between the column and citrus peel components, yet the contrasts in the SEC chromatograms shown in Fig. 3 clearly illustrate the differences in the relative molecular weight profiles in the retentate and filtrate and demonstrate that the elevated HPLC baselines of retentates are likely due to higher molecular weight cell wall components.

**Figure 4:**
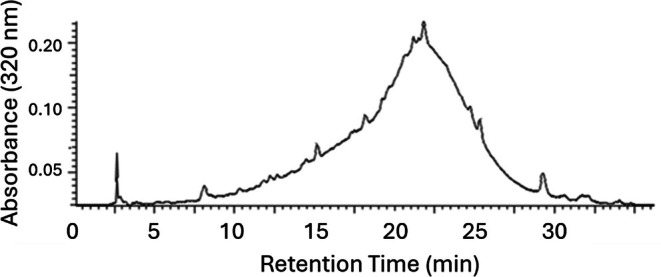
Early‐eluting LH20 column chromatography peak (fractions 1–12) of processed lemon peel extract. Subsequent fractions (13–22 and 23–29) also showed flavonoid‐free baseline chromatograms. Similar results were obtained using the methanol‐soluble component of processed orange peel extract and of commercial orange peel molasses.

Studies of the baseline materials were further made using LH20 column chromatography to fractionate the baseline components according to molecular size. In these studies, methanol was used as the eluting solvent, and by doing so minimized the hydrophobic interactions between the LH20 resin and the test compounds, and thus created conditions for separations based mainly on molecular weights. The use of methanol as the eluting solvent necessitated the dissolution of the powdered extracts with methanol, which as a result, effectively precipitated much of the pectin and free sugars from these extract samples, and this significantly simplified the subsequent chromatographic separations and spectroscopic analyses. LH20 chromatography of the methanol‐soluble peel components of the processed lemon and orange peel extracts resulted in the resolution of several distinct peaks (Fig. S2), where the HPLC analyses of the earliest eluting LH20 fractions showed only baseline materials and appeared free of most of the lower molecular weight compounds (Fig. 4). Similar results were obtained using the methanol‐soluble component of processed orange peel extract and of commercial orange peel molasses.

**Figure 5:**
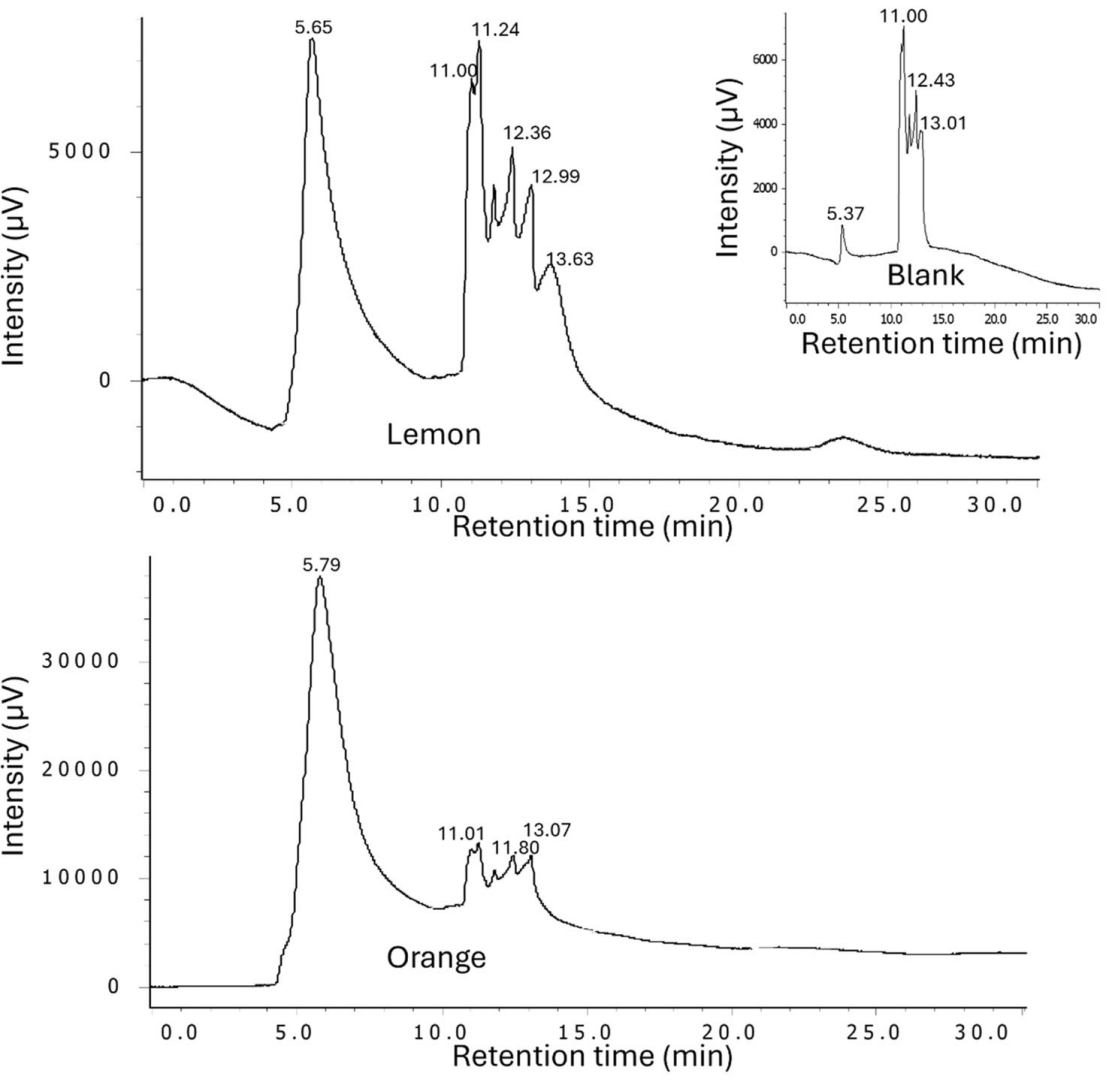
SEC of LH20 baseline fractions of lemon (top) and clarified diluted orange peel molasses (bottom) The background chromatogram is shown in the insert.

SEC measurements, as described earlier, were taken with the early‐eluting LH20 baseline fractions from lemon peel extract and orange peel clarified molasses (Fig. 5). The elution times of the main peaks in these column fractions (5.79 min for orange and 5.65 min for lemon) were similar, indicating molecular weights between 30 to 60 kDa, consistent with those of the earlier studied ultrafiltration retentates (Fig. 3).

### Spectroscopy

The UV spectra of these early‐eluting baseline fractions from both orange and lemon peel extracts showed wavelength maxima near 275 and 320 nm (Fig. S3). These absorption maxima indicate the presence of substituted aromatic phenyl and phenylpropenyl ring structures, notably similar to those in lignin and suberin.^10^, ^33^, ^34^, ^35^

**Figure 6:**
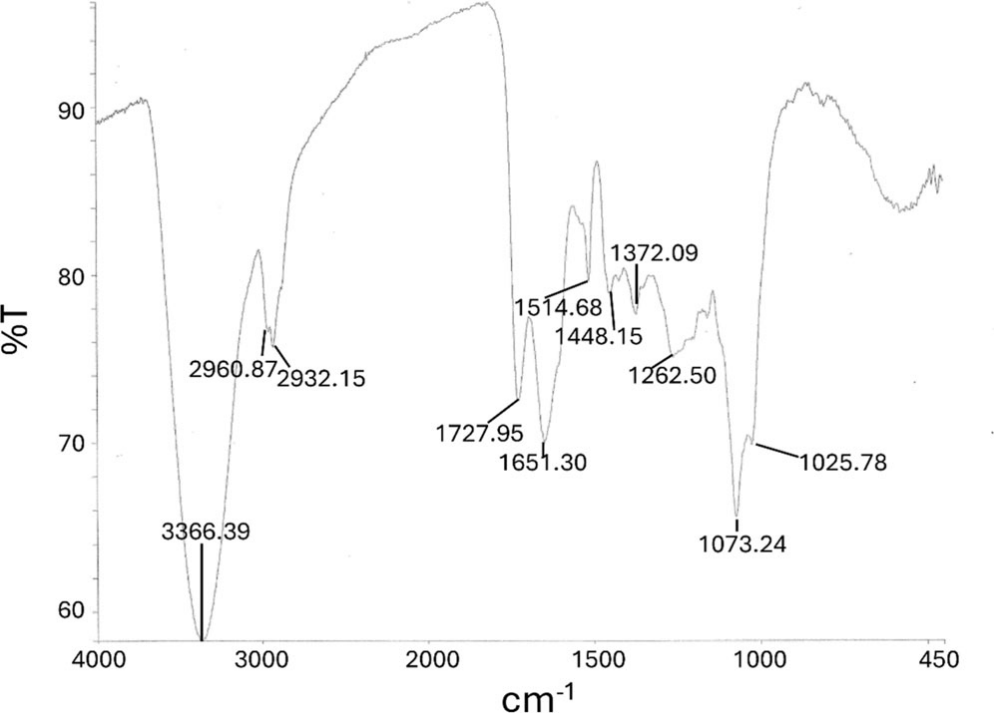
FTIR of the methanol‐soluble portion of the initial LH20 column baseline fraction of orange peel extract.

The chemical characterizations of these LH20 column baseline fractions and ultrafiltration retentates and filtrates were further analyzed by FTIR spectroscopy. The FTIR spectrum of the LH20 column baseline fraction obtained from the orange peel extract, shown in Fig. 6, is similar to that of the LH20 column baseline fraction obtained with the processed lemon peel extract (data not shown). Importantly, close similarities are noted between these spectra and those previously measured for suberin from a variety of other plant sources.^15^, ^17^ The vibrations in Fig. 6 include: 3366 cm^−1^ (broad) O–H stretch (hydroxyls); 2960, 2932 cm^−1^ C–H stretch (long chain alkanes); 1727 cm^−1^ C=O stretch (esters); 1651 cm^−1^ C=O (conjugated carbonyls, amides, water); 1604 cm^−1^ C=C (aromatic phenyls); 1514 cm^−1^ C=C (conjugated alkenes); 1448 and 1372 cm^−1^ C–H bending (aliphatic chains); 1262 cm^−1^ C–O–C (esters); 1073–1076 cm^−1^ C–O (alcohols, sugars), and 600–400 cm^−1^ broad (water). Previous chemical and spectroscopic analyses of suberin of other plants have led to its description in hypothetical models,^14^, ^16^, ^18^, ^36^ all of which include the earlier‐mentioned functional groups.

When the LH20 baseline fractions were dried to a free‐flowing powder, this material occurred as two distinct subfractions; the major portion occurred as a methanol soluble material, while a minor portion (~20%) remained methanol insoluble. The methanol soluble portion, when analyzed by FTIR, exhibited a FTIR spectrum closely similar to that shown in Fig. 6. In contrast, the FTIR spectrum of the methanol‐insoluble portion (Fig. S4) showed no carbonyl ester vibration at 1732 cm^−1^, but rather showed two prominent vibrations at 1645 and 1537 cm^−1^, features of which suggest the presence of protein as the main component of this methanol‐insoluble material.^37^, ^38^ As described in the ‘Materials and methods’ section, the bulk of the pectin had already been removed from this peel extract by passage through anion exchange resin.

**Figure 7:**
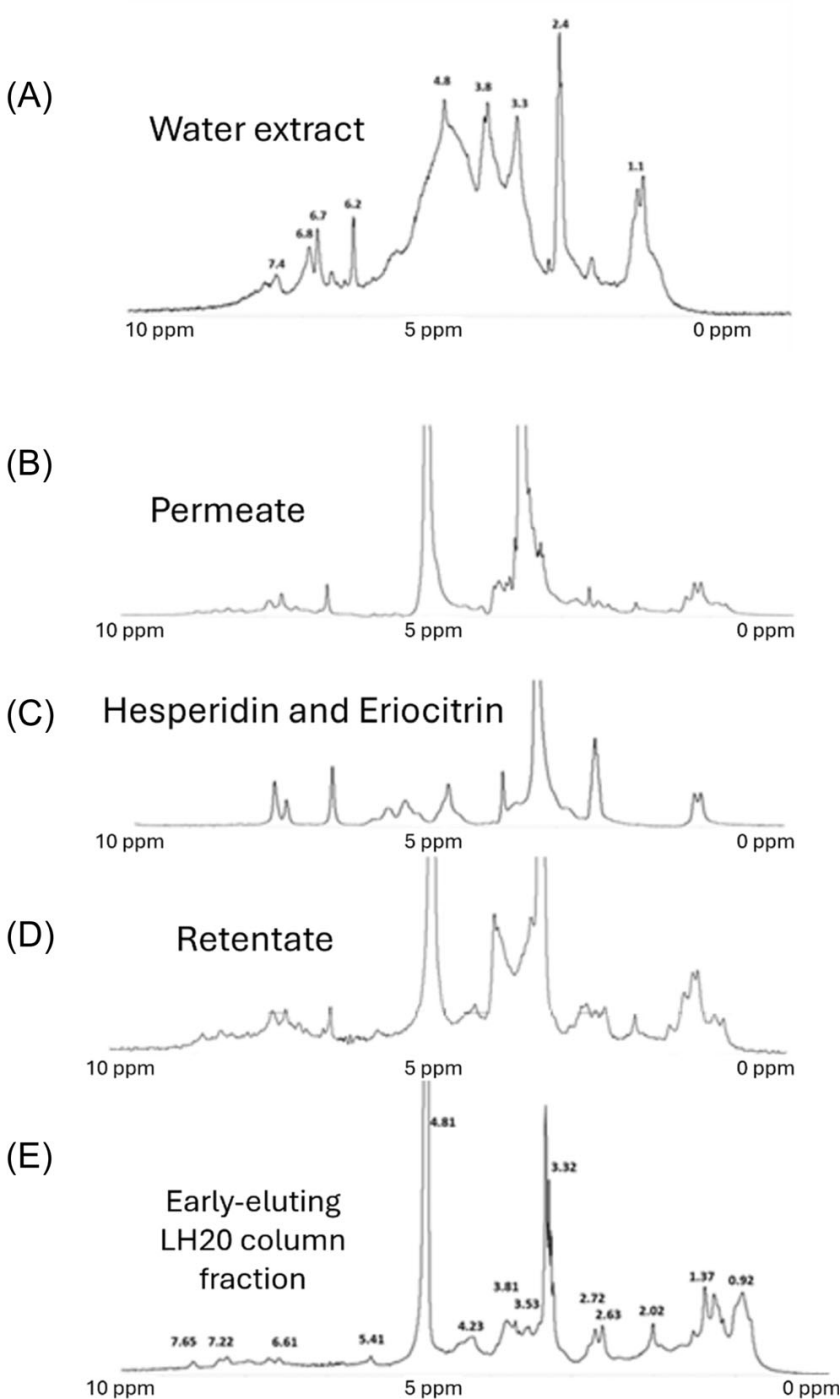
^1^H‐NMR of original processed lemon peel water extract (A). ^1^H‐NMR of permeate of processed lemon peel water extract (B), equimolar hesperidin and eriocitrin in DMSO‐*d*_6_ (C), and retentate of processed lemon peel water extract (D). ^1^H‐NMR of a lemon peel early‐eluting LH20 column fraction in which lemon flavonoids are absent (E).

To further test the hypothesis that the citrus baseline materials may be largely composed of suberin, NMR spectra were measured for a number of the early‐eluted LH20 column fractions. Previous studies published elsewhere have shown NMR spectroscopy is capable of providing detailed information of structural characteristics of suberin.^14^, ^39^, ^40^, ^41^, ^42^ Carbon‐13 (^13^C) NMR has shown evidence of aromatic rings, methoxyl groups, conjugated hydroxycinnamic acids, methylenes, alkanedionic acids, alkanoic acids, alcohols, carboxylic acids and esters, and long chain alkanes.^18^ In our current study ^1^H‐NMR (60 MHz) spectroscopy was used to compare the chemical compositions of the retentates and permeates of the processed citrus peel extracts. With an emphasis on the aromatic proton region, ^1^H‐NMR spectra of the original lemon peel extract show resonances indicative of the main lemon flavonoids, hesperidin and eriocitrin, as well as of other aromatic proton resonances extending between 6.3 and 8.0 ppm (Fig. 7(A)). These latter resonances appear to be similar to those previously reported for suberin and lignin, where they have been attributed to *p*‐coumarate and *p*‐hydroxyphenyl structural units.^43^, ^44^, ^45^ In a similar manner, the ^1^H‐NMR spectrum of the methanol‐soluble portion of the permeate obtained from the lemon peel extract (Fig. 7(B)) showed major contributions from eriocitrin and hesperidin, the two main flavonoids in lemon peel. As confirmation, an equimolar mixture of hesperidin and eriocitrin showed that both compounds have nearly overlapping low‐resolution ^1^H‐NMR spectra (Fig. 7(C)). The spectrum of the methanol portion of the retentate (Fig. 7(D)) shows a number of similarities to the permeate, including proton resonances of residual amounts of hesperidin and eriocitrin. However, there are additional proton resonances between 0.8 and 5 ppm that are likely due to a variety of other peel components (discussed later). The retentate spectrum also appears to show a much larger relative total integrated aromatic proton resonance area between 6.0 and 8.3 ppm, where, as stated earlier, most of this integrated peak area is likely due to *p*‐coumarate and *p*‐hydroxyphenyl structural units of suberin and possibly to unknown smaller amounts of lignin.

In agreement with the earlier‐discussed results for the processed lemon peel ultrafiltration fractions is the ^1^H‐NMR spectrum of the first early‐eluting LH20 column fraction of the processed lemon peel aqueous extract (Fig. 7(E)). The proton resonance values for this sample are listed in Table 1, column B. The broad proton resonances between 6.5 and 8.3 ppm in this sample, as well as in the lemon peel ultrafiltration retentate and the early‐eluting LH20 column fraction obtained from clarified orange peel molasses, columns C and A of Table 1, respectively, all support the presence of diverse populations of substituted aromatic rings. Also evident are the diverse populations of substituted alkanes suggested by the resonances between 0.8 and 1.7 ppm, the allylic protons at 2.0 ppm, primary and secondary alcohols between 3.6 and 4.6 ppm, and possibly of protons adjacent to carbonyls shown by the resonances near 2.3 and 4.3 ppm. The strong similarities between the spectra of the LH20 column fractions Table 1, columns A and B and the ^1^H‐NMR spectrum measured for the 3 kDa retentate of the processed lemon peel retentate (Table 1, column C) are important, because as discussed earlier, this retentate was obtained using a method separate from that using LH20 chromatography. Notable also are the similarities between these ^1^H‐NMR spectra and that of authentic *Quercus suber* cork suberin (Table 1, column D), although there are slight differences due to the spectra in this study being collected in DMSO‐*d*_6_ and the spectrum of cork suberin being collected in deuterated chloroform (CDCl_3_).

**Table 1:** Proton‐nuclear magnetic resonance (^1^H‐NMR, ppm) for: (column A) early‐eluting LH20 column fraction of solids derived from water‐diluted and clarified commercial orange peel molasses in dimethyl sulfoxide (DMSO), (column B) early‐eluting LH20 column fraction of processed aqueous lemon peel extract in DMSO, (column C) 3 kDa ultrafiltration retentate of clarified processed orange peel molasses in DMSO, and (column D) isolated *Quercus suber* cork suberin in deuterated chloroform (CDCl_3_)^17^

Orange LH20 (column A)	Lemon LH20 (column B)	Lemon retentate (column C)	Cork suberin (column D)
0.81	0.8	0.8	0.60–1.0 aliphatic methyl
0.92	0.92	0.92	0.60–1.0 aliphatic methyl
	1.15	1.15	1.0–1.7 aliphatic methylene
1.22	1.27	1.22	1.0–1.7 aliphatic methylene
1.32	1.37	1.34	1.0–1.7 aliphatic methylene
1.53	1.52	1.53	1.0–1.7 aliphatic methylene
2.01	2.02	2.01	2.0 allylic proton
2.47	2.63	2.42–2.65	2.28 CH_2_COO
	2.72		2.28 CH_2_COO
2.75	2.79		2.28 CH_2_COO
3.19–3.45	3.32 broad	3.3 (multiple)	3.7–3.9 aromatic methoxyl
3.46	3.53	3.42	3.7–3.9 aromatic methoxyl
3.5	3.69		3.63 primary alcohols
3.77, 3.93	3.81	3.92	3.63 primary alcohols
4.32	4.23	4.19	3.9 COOCH_3_
4.45	4.35		4.55 secondary alcohols
4.8	4.7	4.79	4.55 secondary alcohols
5.39–5.80	5.47^a^	5.54	5.32 vinylic protons
6.15	6.61^a^	6.18; 6.27; 6.60	5.4–6.4 phenylpropanoid vinyl
6.6	6.72^a^	6.78	6.4–8.0 aromatic protons
6.96	6.98^a^	6.96	6.4–8.0 aromatic protons
	7.22		6.4–8.0 aromatic protons
7.3	7.32^a^	7.3	6.4–8.0 aromatic protons
7.69	7.65^a^	7.67; 7.91	6.4–8.0 aromatic protons
5.8–8.3^a^ broad	5.8–8.3^a^ broad		6.4–8.0 aromatic protons

^a^ Also in LH20 column fractions 40–47.

## CONCLUSION

In this study the phenomenon of the broad elevated HPLC baselines of water extracts of citrus peels is illustrated as well as the enrichment, recovery and isolation of the materials responsible for these baselines by ultrafiltration and preparative and analytical SEC. The results of these enrichment and isolation techniques indicated that these baseline materials were composed of broad populations of diverse unidentified high molecular weight chemical constituents. This led to further characterizations of these baseline materials by several spectroscopic methods.

The overall results reported in this study suggest that the components responsible for the elevated HPLC baselines from extracts of lemon peel, orange peel, and orange peel molasses are mainly suberin. In addition to these main components, lower molecular weight polymers were also detected in isolated suberin. As discussed earlier, SEC analyses of the 3 kDa lemon and orange peel extract retentates provided evidence of high molecular weight components as the main constituents in these samples. Hence, these similarities as well as those of the average molecular weights of the citrus baseline materials compared to those of suberin lend support to our hypothesis that suberin is a significant part of these baseline materials and are likely significant portions of many commercial citrus peel extracts.

Future work could include the identification of suberin monomers via gas chromatography–mass spectrometry (GC–MS) analysis following a depolymerizing methanolysis providing additional validation of the presence of suberin. Additionally, since aqueous extracts are utilized as flavonoid‐enriched citrus peel supplements, future biological tests of these flavonoid‐enriched citrus peel components could consider possible contributions of suberin to these examinations. Subsequent to testing for the presence of suberin, future experiments could turn towards understanding any potentially occurring health benefits of this material. Although it needs to be validated in the future, methods described herein suggest that obtaining higher purity flavonoid fractions may be possibly achieved by the removal of suberin and other higher molecular weight peel components by ultrafiltration.

## Supporting information


**Figure S1.** Polyethylene glycol molecular weight standards on TSK gel Alpha‐3000 SEC 90 kDa molecular weight cut‐off. Standards and samples run in water at 1.0 mL min^−1^. SEC polyethylene glycol standards (Agilent EasiVial PEG/PEO (P.N. 2080‐0201)) were used to estimate the molecular weight ranges of chemical components in peel extracts.
**Figure S2.** LH20 column chromatogram of methanol soluble clarified orange peel molasses.
**Figure S3.** UV spectrum of the early‐eluting LH20 ‘baseline’ fraction of orange peel extract.
**Figure S4.** FTIR of water‐soluble portion of early‐eluting LH20 column baseline fraction.

## Data Availability

The data that support the findings of this study are available from the corresponding author upon reasonable request.
