# Supplementary material for: Suberin. A component of citrus peel extracts
Source: J Sci Food Agric. 2025 Aug 8;105(15):8392–9. doi: 10.1002/jsfa.70075 (PMC12595398; doi:10.1002/jsfa.70075)
Supplement: Supplementary file 1 — Figure S1. Polyethylene glycol molecular weight standards on TSK gel Alpha‐3000 SEC 90 kDa molecular weight cut‐off. Standards and samples run in water at 1.0 mL min−1. SEC polyethylene glycol standards (Agilent EasiVial PEG/PEO (P.N. 2080‐0201)) were used to estimate the molecular weight ranges of chemical components in peel extracts. Figure S2. LH20 column chromatogram of methanol soluble clarified orange peel molasses. Figure S3. UV spectrum of the early‐eluting LH20 ‘baseline’ fraction of orange peel extract. Figure S4. FTIR of water‐soluble portion of early‐eluting LH20 column baseline fraction. [file JSFA-105-8392-s001.pptx]

## Slide 1
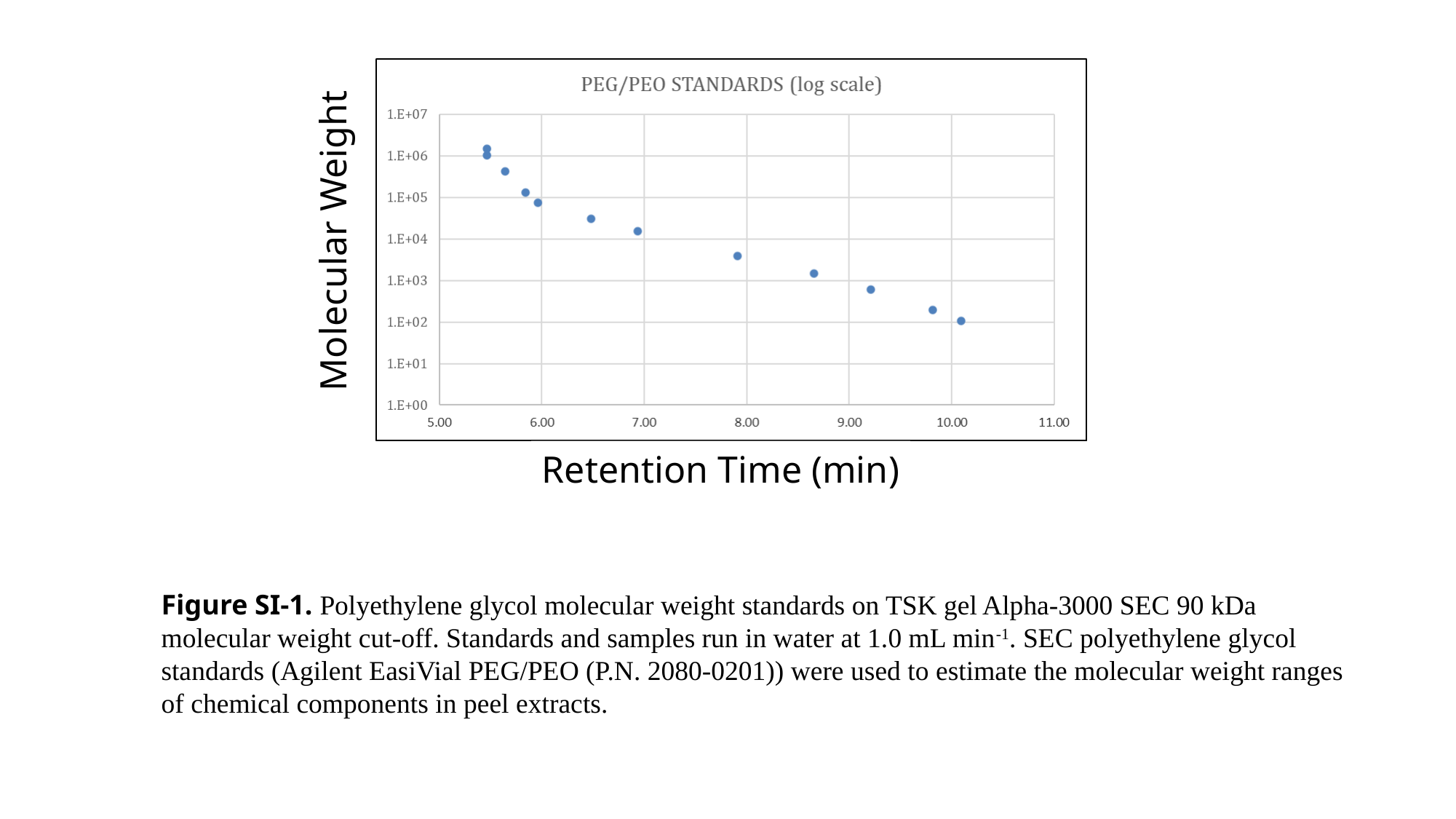

Molecular Weight
Retention Time (min)
Figure SI-1. Polyethylene glycol molecular weight standards on TSK gel Alpha-3000 SEC 90 kDa molecular weight cut-off. Standards and samples run in water at 1.0 mL min-1. SEC polyethylene glycol standards (Agilent EasiVial PEG/PEO (P.N. 2080-0201)) were used to estimate the molecular weight ranges of chemical components in peel extracts.

## Slide 2
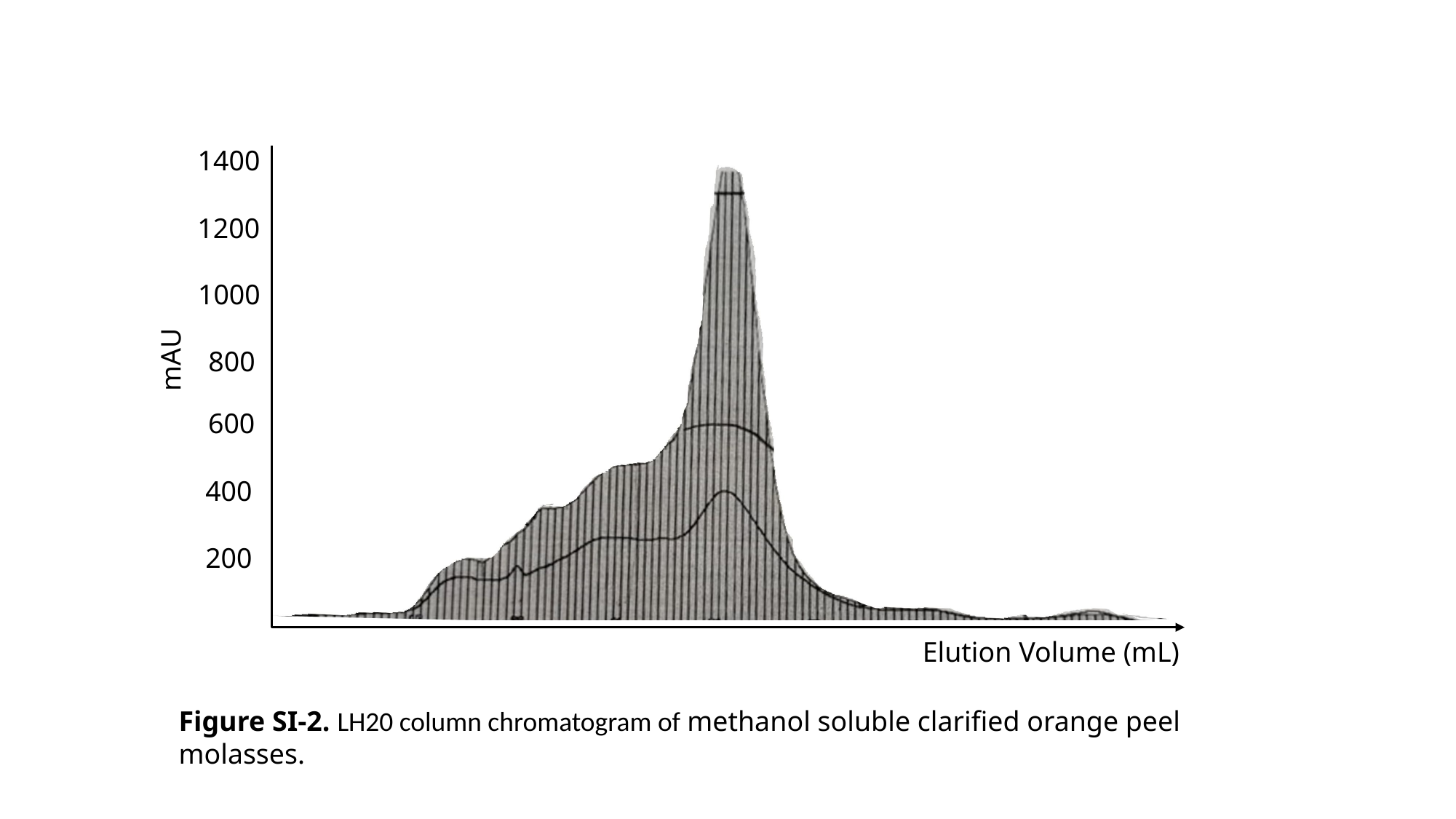

1400
1200
1000
mAU
800
600
400
200
					Elution Volume (mL)
Figure SI-2. LH20 column chromatogram of methanol soluble clarified orange peel molasses.

## Slide 3
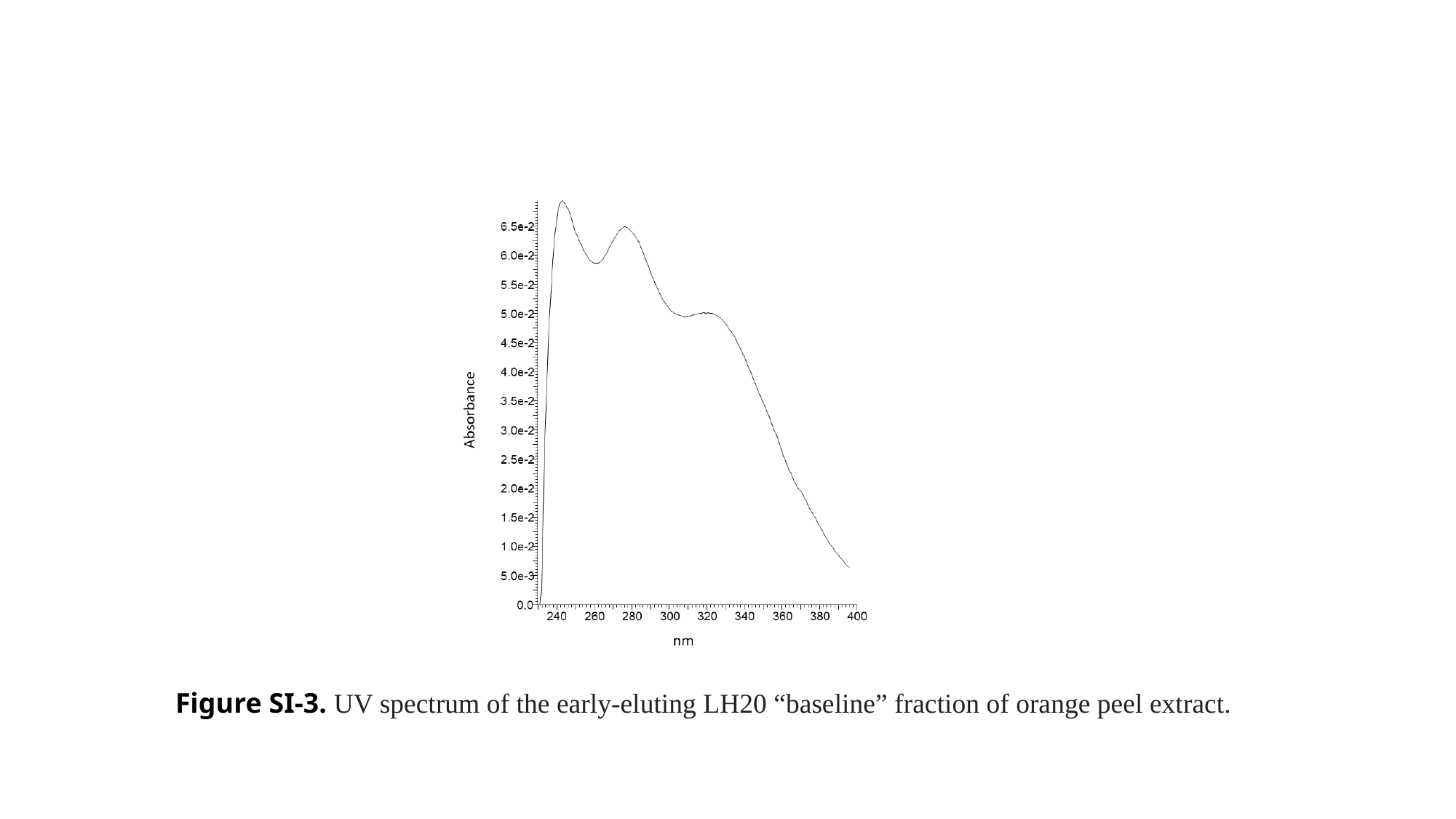

Figure SI-3. UV spectrum of the early-eluting LH20 “baseline” fraction of orange peel extract.

## Slide 4
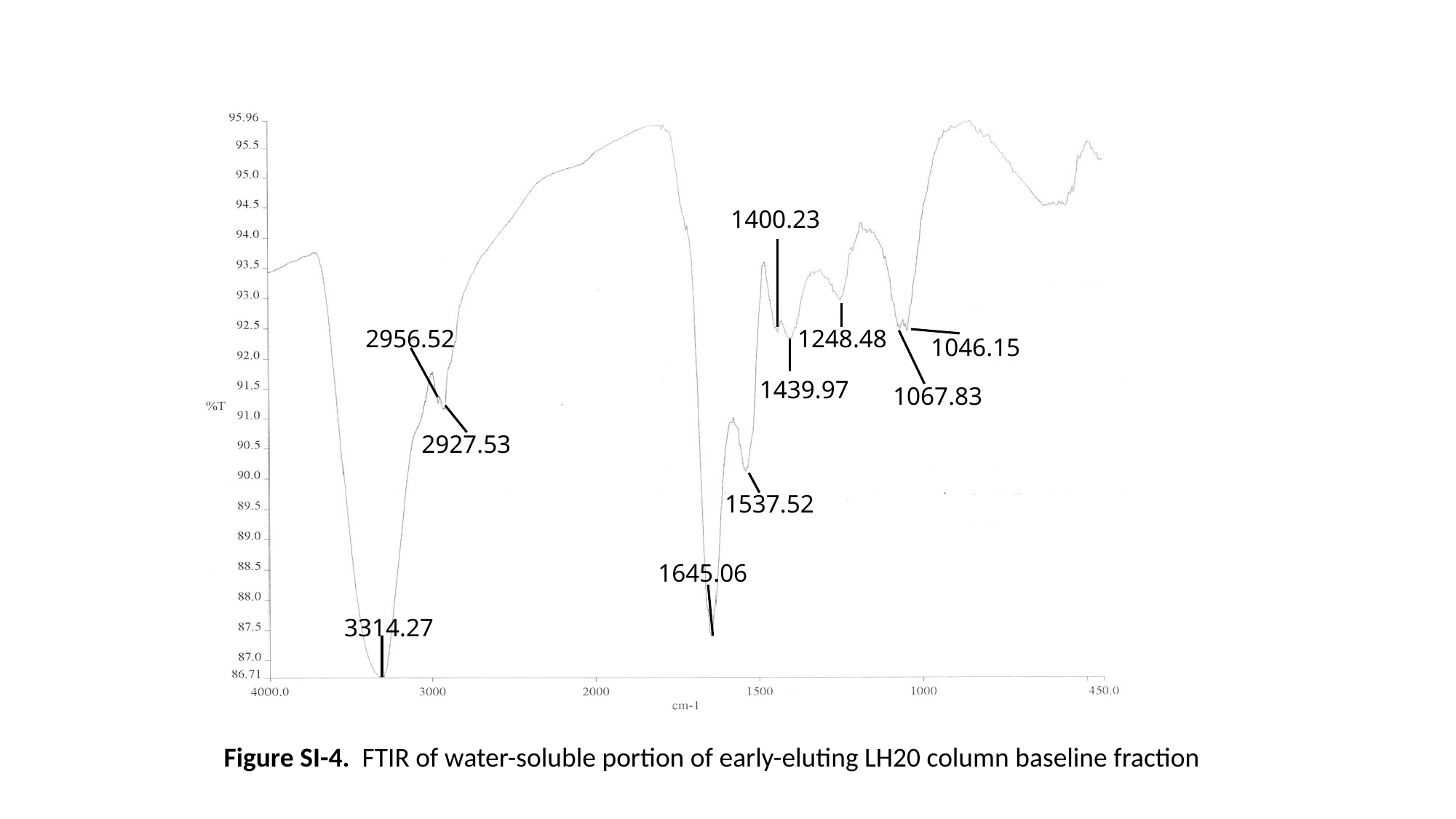

1400.23
2956.52
1248.48
1046.15
1439.97
1067.83
2927.53
1537.52
1645.06
3314.27
Figure SI-4. FTIR of water-soluble portion of early-eluting LH20 column baseline fraction
